# Unsupervised Cluster Analysis in Patients with Cardiorenal Syndromes: Identifying Vascular Aspects

**DOI:** 10.3390/jcm13113159

**Published:** 2024-05-28

**Authors:** Jean-Baptiste de Freminville, Jean-Michel Halimi, Valentin Maisons, Guillaume Goudot, Arnaud Bisson, Denis Angoulvant, Laurent Fauchier

**Affiliations:** 1 Service de Cardiologie-Médecine Vasculaire, Hôpital Trousseau, Centre Hospitalier Regional Universitaire de Tours, 37044 Tours Cedex 9, France; 2 Service de Medecine Vasculaire, Hopital Europeen Georges Pompidou, Assistance Publique Hôpitaux de Paris, Université Paris Cité, 75015 Paris, France; guillaume.goudot@aphp.fr; 3 Néphrologie-Immunologie Clinique, Hôpital Bretonneau, Centre Hospitalier Regional Universitaire de Tours, 37000 Tours, France; halimi@univ-tours.fr (J.-M.H.); valentin.maisons@univ-tours.fr (V.M.); 4 Faculté de Medecine, UMR Inserm University of Tours 1327 ISCHEMIA “Membrane Signalling and Inflammation in Reperfusion Injuries”, 37044 Tours, France; arnaud.bisson@univ-tours.fr (A.B.); d.angoulvant@chu-tours.fr (D.A.); l.fauchier@chu-tours.fr (L.F.); 5 F-CRIN INI-CRCT, 10, Boulevard Tonnellé, 37032 Tours, France; 6 INSERM U1246 SPHERE, Universities of Nantes and Tours, 37044 Tours, France; 7 INSERM U970 PARCC, Université Paris Cité, 75015 Paris, France; 8 Service de Cardiologie, Centre Hospitalier Universitaire Trousseau et Faculté de Médecine, 37044 Tours, France

**Keywords:** cardiorenal syndrome, chronic kidney disease, heart failure

## Abstract

**Background/Objectives**: Cardiorenal syndrome (CRS) is a disorder of the heart and kidneys, with one type of organ dysfunction affecting the other. The pathophysiology is complex, and its actual description has been questioned. We used clustering analysis to identify clinically relevant phenogroups among patients with CRS. **Methods**: Data for patients admitted from 1 January 2012 to 31 December 2012 were collected from the French national medico-administrative database. Patients with a diagnosis of heart failure and chronic kidney disease and at least 5 years of follow-up were included. **Results**: In total, 13,665 patients were included and four clusters were identified. Cluster 1 could be described as the vascular–diabetes cluster. It comprised 1930 patients (14.1%), among which 60% had diabetes, 94% had coronary artery disease (CAD), and 80% had peripheral artery disease (PAD). Cluster 2 could be described as the vascular cluster. It comprised 2487 patients (18.2%), among which 33% had diabetes, 85% had CAD, and 78% had PAD. Cluster 3 could be described as the metabolic cluster. It comprised 2163 patients (15.8%), among which 87% had diabetes, 67% dyslipidemia, and 62% obesity. Cluster 4 comprised 7085 patients (51.8%) and could be described as the low-vascular cluster. The vascular cluster was the only one associated with a higher risk of cardiovascular death (HR: 1.48 [1.32–1.66]). The metabolic cluster was associated with a higher risk of kidney replacement therapy (HR: 1.33 [1.17–1.51]). **Conclusions**: Our study supports a new classification of CRS based on the vascular aspect of pathophysiology differentiating microvascular or macrovascular lesions. These results could have an impact on patients’ medical treatment.

## 1. Introduction

Cardiorenal syndrome (CRS) is defined as a disorder of the heart and kidneys, with one type of organ dysfunction affecting the other [1]. 

**Figure 1:**
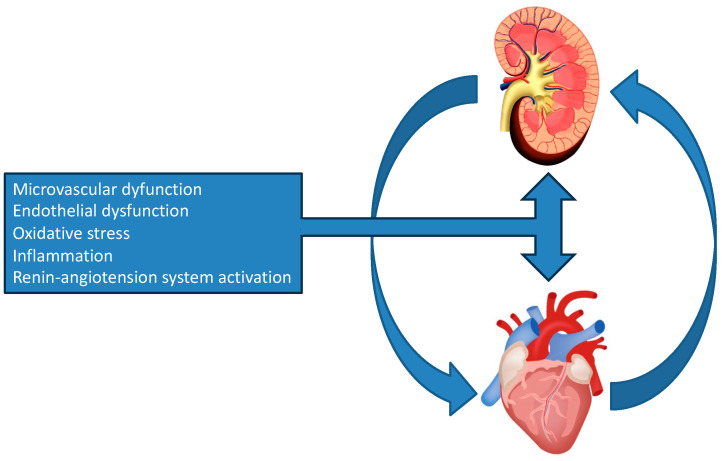
Cardiorenal syndrome connection.

The most used classification of CRS distinguishes five types [2,3,4] and is based on the chronology of cardiac and renal events. However, this classification presents several issues. First, we recently found, in a large nationwide cohort study, that the chronology between kidney and heart events was not associated with different prognoses. Moreover, there was no synergism between the kidney and heart, which supports the fact that CRS is the consequence of a shared pathophysiology among these two organs, rather than one organ’s dysfunction caused by the other [5]. These pathological pathways have been studied and described and include microvascular and endothelial dysfunction, the activation of the sympathetic and renin–angiotensin–aldosterone (RAA) systems, alterations in nitric oxide bioavailability, or inflammation [6,7], and a consistent hypothesis is that the major common consequence is fibrosis [8]. The role of microvascular dysfunction in the development of HF and CKD is well documented, although the mechanism differs between HF with preserved ejection fraction (HFpEF) and that with reduced ejection fraction (HFrEF) [9,10,11,12]. In brief, in the first case, cardiac remodeling involves inflammation and endothelial dysfunction, whereas, in HFrEF, remodeling is predominantly the consequence of cardiomyocyte death. Regarding the kidneys, among the potential mechanisms, a central venous pressure increase and a reduction in renal blood flow lead to decreased nitric oxide (NO) production and a vicious circle involving sympathetic and RAA system activation, finally leading to fibrosis [8,9,13] (Figure 1).

Moreover, the acute types (1 and 3) and type 5 refer to specific situations with known systemic consequences and should be distinguished. This view was defended by Zoccali et al. in a recent position paper [14]. Another view is cardiovascular–kidney–metabolic syndrome, an entity recently recognized by the Amercian Heart Association, expanding the classical cardiorenal syndrome and advocating for a more holistic view [15]. While type 2 and type 4 (chronic CRS) should be redefined outside any chronological considerations, CRS may refer, in acute situations, to functional acute kidney injury (AKI) in conditions of decompensated heart failure, improving after the treatment of congestion. This definition of CRS, which would refer to some of the type 1 cases, is, to our knowledge, the most largely applied in clinical practice and in the literature [16,17,18]. In this study, we aimed to explore the fact that, rather than the clinical presentation, the pathophysiological mechanism involved could be used to better classify these patients and lead to optimized treatment [8]. 

Cluster analysis is an unsupervised machine learning method that categorizes complex entities without investigators’ supervision by segregating samples into homogenous groups [19,20,21]. Unsupervised analysis can have great value, as it allows one to explore data without a priori knowledge. 

In this study case, we hypothesized that unsupervised clustering would corroborate the pathophysiological classification of CRS by identifying clinically relevant phenogroups related to known pathophysiological pathways.

## 2. Materials and Methods

### 2.1. Study Design

This longitudinal cohort study utilized the national hospitalization database, which covers hospital care for the entire French population. Data for patients admitted from 1 January 2012 to 31 December 2012 were collected from the national medico-administrative PMSI database (“Programme de Médicalisation des Systèmes d’Information”), a medicalized information system program inspired by the US Medicare system. This program, implemented in 2004, records hospital medical activity in a database, ensuring anonymity, and encompasses over 98% of the French population (approximately 67 million people) from birth (or immigration) to death (or emigration). 

The hospitalization details are encoded in a standardized dataset, including age, sex, hospital stay duration, admission and discharge dates, mode of discharge, pathologies, and procedures. The medical information collected routinely comprises principal and secondary diagnoses based on the International Classification of Diseases, Tenth Revision (ICD-10). All medical procedures are also recorded using the national nomenclature, Classification Commune des Actes Medicaux (CCAM). The reliability of the PMSI data has been previously assessed [22], and this database has been effectively used in previous studies related to cardiovascular or cerebrovascular conditions [5,23].

The study was conducted retrospectively, and, as the patients were not involved in its conduct, there was no impact on their care. Ethical approval was not required, as all data were anonymized. The French Data Protection Authority granted access to the PMSI data. The procedures for data collection and management were approved by the Commission Nationale de l’Informatique et des Libertés (CNIL), the independent national ethical committee protecting human rights in France, which ensures that all information is kept confidential and anonymous, in compliance with the Declaration of Helsinki (MR-005 registration number 0415141119).

### 2.2. Patient Selection

This study was based on patients aged 18 years and over, admitted to a French hospital between 1 January 2012 and 31 December 2012, with a diagnosis of HF and CKD. The analysis focused on patients with at least 5 years of follow-up or who died during follow-up. The patients’ medical history in the 2 years before their hospitalization was collected. Patients with CRS were identified as patients with a diagnosis of both HF and CKD. For the cluster analysis, a random sample of 50% of these patients was finally included in this study. 

### 2.3. Collected Data

Patient information (demographics, comorbidities, medical history, and events during hospitalization or follow-up) was described using data collected in the hospital records. For each hospital stay, combined diagnoses at discharge were obtained. Each variable was identified using ICD-10 codes (Appendix A Table A1: ICD-10 codes).

### 2.4. Outcomes

We aimed to evaluate the incidence of all causes of death, cardiovascular death, rehospitalization for HF, myocardial infarction, ischemic stroke, and KRT (defined by dialysis or renal transplantation) (Appendix A Table A1: ICD-10 codes).

The endpoints were evaluated with follow-up starting from the first hospitalization until the date of each specific outcome or the date of last news in the absence of the outcome. Information on outcomes during follow-up was obtained by analyzing the PMSI codes for each patient. We focused on the components of Major Adverse Renal and Cardiac Events (MARCE) [24,25], namely all-cause death, cardiovascular death, hospitalization for heart failure, myocardial infarction, stroke, and renal replacement therapy, with the exception of acute kidney injury, which were identified by using their respective ICD-10 or procedure codes. Cardiovascular death was determined based on the primary diagnosis during hospitalization resulting in death (ICD-10 codes: cardiovascular death—I00-I99). Rehospitalization was considered to be attributable to heart failure when heart failure was recorded as the main diagnosis.

### 2.5. Cluster Analysis

**Figure 2:**
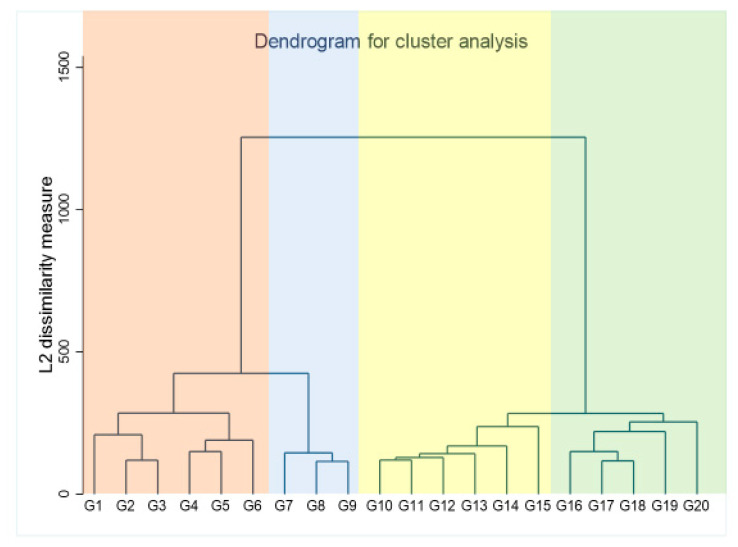
Dendrogram generated by hierarchical clustering process showing the CRS clusters. The dendrogram graph is a visual representation of the hierarchical clustering process. Vertical lines are clusters that are joined together, and the position of the line on the scale indicates the distance at which the clusters are joined. Clusters are identified by different colors.

Unsupervised cluster analysis using the hierarchical clustering method was used to identify homogenous phenotypic subgroups of patients with CRS, without prior knowledge of the outcomes. All baseline clinical variables were used for the clustering process (Table 1). Agglomerative hierarchical clustering was performed, using Ward’s method, and the squared Euclidian distance was used for the variables of interest. Agglomerative hierarchical clustering is based on a ‘bottom-to-top’ approach where the clustering begins with a single patient, who is then grouped with another based on similarities regarding the specified clinical variables. The dendrogram (Figure 2) displays the clustering process with the vertical lines representing the various clusters and the distance between the clusters equating to the sum of the squared differences within all clusters. Small values of the distance indicate that the merged clusters are similar, and large values indicate the combination of 2 dissimilar (heterogeneous) clusters. The determination of the number of clusters was not prespecified. Two-, three-, and four-cluster models were examined. The four-cluster model formed much clearer patterns than the three-cluster model and was therefore used in this study.

### 2.6. Statistical Analysis

Qualitative variables were described as frequencies and percentages and quantitative variables as means (SDs). Comparisons were made using χ2 tests for categorical variables and the Student *t* test or nonparametric Kruskal–Wallis test, as appropriate, for continuous variables.

The 5-year yearly incidence of all-cause death, cardiovascular death, rehospitalization for HF, myocardial infarction, ischemic stroke, and KRT was calculated. Unadjusted and multivariable-adjusted Cox analyses were used to estimate the associations between clusters and clinical outcomes, and the results were expressed as hazard ratios (HR) and 95% confidence intervals (95% CI). Incidence rates (IR) were also reported in each cluster. Parameters associated with the risk of death, cardiovascular death, myocardial infarction, new episodes of HF, ischemic stroke, and KRT were used as covariates in the multivariable models.

## 3. Results

### 3.1. Baseline Characteristics of Patients

A total of 13,665 patients were included in this study, among whom 57% were men and 77% were older than 75 years. Most patients had hypertension (82%), 44% had diabetes, 34% had dyslipidemia, 24% had obesity, and 8% were active smokers. Peripheral artery disease (PAD) was present in 39% of the patients. 

Regarding associated cardiac conditions, the majority (56%) had coronary artery disease, 23% had dilated cardiomyopathy, 53% had atrial fibrillation, and 25% had valvular heart disease.

**Figure 3:**
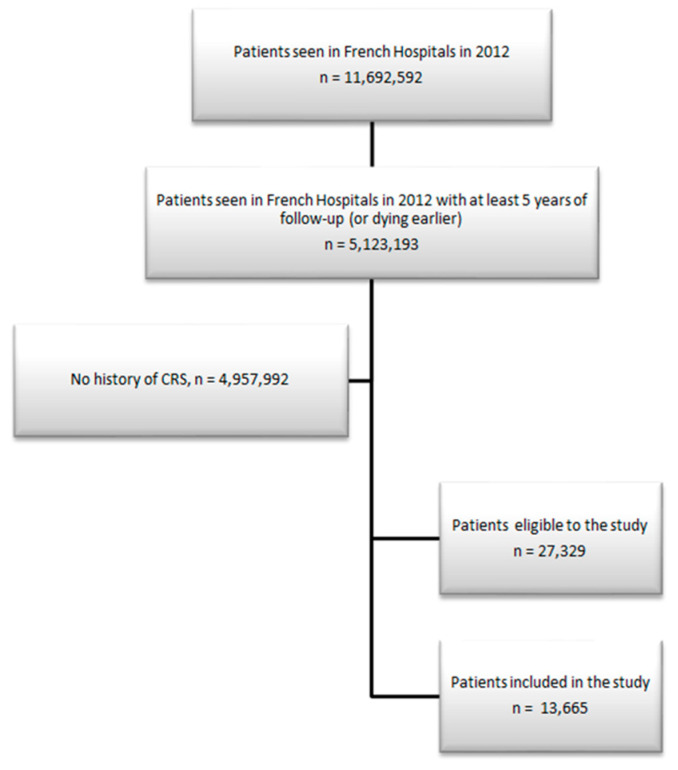
Flow chart.

The characteristics of all patients are detailed in Table 1 and Figure 3.

### 3.2. Cluster Analysis

Based on the hierarchical cluster analysis, four different clusters were identified (central illustration).

**Figure 4:**
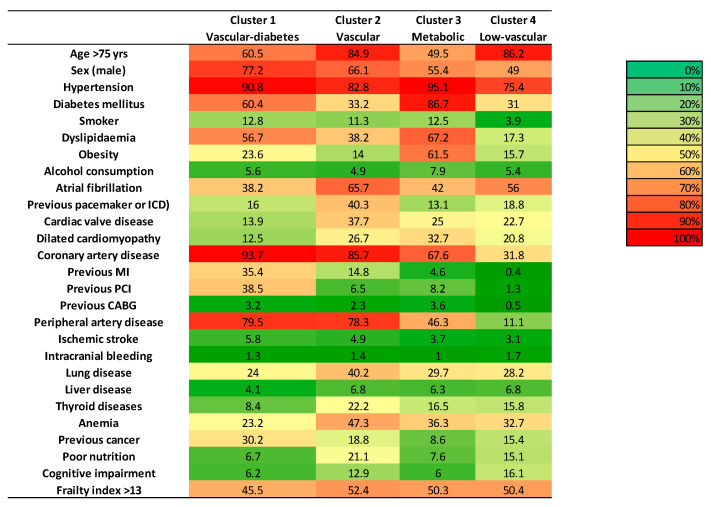
Heatmap of baseline characteristics of patients with CRS according to patient clusters.

The patients’ characteristics per cluster are shown in Table 1 and Figure 4.

#### 3.2.1. Cluster 1

Cluster 1 comprised 1930 patients (14.1% of the population). It could be described as the vascular–diabetes cluster. The mean age was the second lowest (76 years old) and 60% of the patients were over 75 years old. However, 91% of the patients had hypertension, 60% had diabetes, 94% had coronary artery disease, and 80% had PAD. Atrial fibrillation was less frequent than in the other clusters (38%).

#### 3.2.2. Cluster 2

Cluster 2 comprised 2487 patients (18.2% of the population). It could be described as the vascular cluster. The mean age was the highest (82 years old) and 85% of the patients were over 75 years old; 66% of the patients had atrial fibrillation and 38% of the patients had valvular heart disease. Only 33% of the patients had diabetes, but 78% had PAD. Anemia was also the most frequent in this group (47.3%).

#### 3.2.3. Cluster 3

Cluster 3 comprised 2163 patients (15.8% of the population). It could be described as the metabolic cluster. The mean age was 74 years old and only 50% of the patients were over 75 years old. Diabetes was present in almost 87% of the patients, dyslipidemia in 67%, and obesity in 62%, whereas only 46% had PAD.

#### 3.2.4. Cluster 4

Cluster 4 comprised 7085 patients (51.8% of the population). It could be described as the low-vascular cluster. There were more women than in the other clusters (51%). The mean age was high (82 years old) and 86% of the patients were over 75 years old. These patients had fewer vascular comorbidities than in the other clusters: 31% of the patients had diabetes, 32% had coronary artery disease, 11% had PAD, 56% had atrial fibrillation, 17% had dyslipidemia, and 16% were obese.

### 3.3. Clinical Outcomes

#### 3.3.1. Univariate Analysis

The results of the survival analysis are shown in Table 2. In the univariate analysis, the metabolic cluster (cluster 3) was associated with a lower risk of all-cause death (HR: 0.89 [0.82–0.95]), but not cardiovascular death (HR: 0.92 [0.82–1.05]), than in the vascular–diabetes cluster (cluster 1), and with a higher risk of dialysis or renal transplantation (HR: 1.31 [1.16–1.49]). The vascular cluster (cluster 2) and the low-vascular cluster (cluster 4) were associated with a higher risk of all-cause death (HR: 1.73 [1.62–1.85] and HR: 1.49 [1.40–1.58], respectively, reference group cluster 1), a higher risk of cardiovascular death (HR: 1.90 [1.69–2.12] and HR: 1.37 [1.24–1.52], respectively), a higher risk of myocardial infarction (HR: 1.58 [1.46–1.70] and HR: 1.28 [1.20–1.36], respectively), and a lower risk of dialysis or renal transplantation (HR: 0.79 [0.68–0.92] and HR: 0.80 [0.71–0.90], respectively).

The risk of myocardial infarction or ischemic stroke was not different between the vascular–diabetes cluster and the other clusters.

#### 3.3.2. Multivariate Analysis

The results of the survival analysis are shown in Table 2. The analyses were adjusted for age and sex.

In the multivariable analyses, the young–metabolic cluster was not associated with a lower risk of death (HR: 1.01 [0.94–1.09]) or cardiovascular death (HR: 1.04 [0.92–1.18]) than the young–vascular–diabetes cluster, but it was still associated with a higher risk of KRT (HR: 1.33 [1.17–1.51]). The vascular cluster and the low-vascular cluster were still associated with a higher risk of death (HR: 1.40 [1.31–1.50] and HR: 1.20 [1.13–1.28], respectively), but only the vascular cluster was associated with a higher risk of cardiovascular death (HR: 1.48 [1.32–1.66]). The risk of dialysis or renal transplantation was not lower in the vascular cluster or the low-vascular cluster than in the vascular–diabetes cluster.

The risk of myocardial infarction or ischemic stroke was still not different between the vascular cluster and the other clusters.

The risk of rehospitalization for heart failure was higher in the vascular cluster, the young–metabolic cluster, and the low-vascular cluster than in the vascular–diabetes cluster.

## 4. Discussion

Our study presents a four-cluster distribution of patients with coupled cardiac and renal involvement, defining CRS. These four clusters correspond to the distribution of patients according to their comorbidities and prognosis, which does not follow the usual CRS classification and thus questions the practical usefulness of this classification and suggests a vascular aspect of pathophysiology in CRS. The objective of this study was to propose a pathophysiological classification of CRS by identifying clinically relevant phenogroups related to known pathophysiological pathways. Its main finding is the identification of four data-driven clusters, which give insights into the different clinical phenotypes of cardiorenal syndromes and can be linked to a known pathophysiology.

The first cluster (14.1% of the population) identified patients with severe vascular damage and frequent diabetes. The second cluster (18.2% of the population) identified patients with severe vascular damage but mostly without diabetes. The third cluster (15.8% of the population) identified patients with metabolic syndrome, diabetes, obesity, dyslipidemia, and fewer macrovascular complications. Finally, the fourth cluster (51.8% of the population) identified patients with fewer comorbidities than in the other clusters, apart from a high rate of atrial fibrillation. This illustrates the variety of clinical phenotypes in cardiorenal syndromes, where the vascular and metabolic history play an important role. Indeed, most comorbidities associated with microvascular dysfunction include diabetes, obesity, dyslipidemia, and hypertension [9,12]. However, these are also risk factors for atherosclerosis and macrovascular lesions. As we have already shown in a previous study, diabetes is a major risk factor for CRS [23]. However, diabetes with (cluster 1) or without (cluster 3) severe macrovascular damage may lead to CRS by different pathophysiological pathways: primary ischemic lesions in the first case and primary microvascular dysfunction in the second case. Moreover, most of our patients (cluster 4) had low vascular damage and few risk factors. We therefore hypothesize that, in these patients, the primary mechanism is microvascular dysfunction, associated with HFpEF, and heart and kidney fibrosis. This is of importance as it may help physicians to better select interventions aimed at preventing clinical events, such as lipid-lowering therapies, anti-hypertensive therapies, or inflammation-modulating therapies.

Of note, anemia is frequent in our population, which is not surprising, and its association with CKD, HF, and CRS is known. The triad of HF, CKD, and anemia is sometimes referred as cardiorenal anemia syndrome [26]. It probably involves hypoxia, nitric oxide secretion, and vasodilation and lowers the blood pressure, which impairs kidney function and can result in sympathetic RAAS activation and renal vasoconstriction and finally fibrosis [27,28,29].

Regarding the impact on the prognoses of these patients, we focused on the components of MARCE, namely death, cardiovascular death, hospitalization for heart failure, myocardial infarction, stroke, and renal replacement therapy, with the exception of acute kidney injury [25]. The risks of death and cardiovascular death seemed to be lower in the vascular–diabetes cluster, even after adjustment for age and sex. On the other hand, the risk of dialysis or renal transplantation was the highest in the metabolic cluster, which was consistent with the high proportion of patients with diabetes and the known impact of cardiovascular risk factors in kidney function decline [1]. The age and sex ratios were very different within the clusters, which may have had a significant impact on the outcomes, even after statistical adjustment. The frailty index was comparable in clusters 2 to 4 and slightly lower in cluster 1, which could explain the lower risk of death in the vascular–diabetes cluster. As expected, macrovascular damage seemed to be associated with a higher risk of death, cardiovascular death, or heart failure (cluster 2). The high proportion of anemia in this cluster may also be related to this poor prognosis.

Environmental or genetic factors could also be involved [30]. Geographical data and the possibility of pollution exposition could shed some light on this question, but, unfortunately, these data were not accessible. The description of CRS based on pathophysiology can greatly impact patients’ care, as individualized medical treatments can be proposed according to the patients’ phenotypes [31].

The strengths of this study are its size and the absence of selection bias because of the exhaustive extraction of all ICD-10 codes from French patients hospitalized in 2012. Limitations include the lack of biological parameters, imaging parameters, and information on drug treatments. Indeed, the elevation of B-type natriuretic peptide, serum creatinine, or more specific ones such as cystatin C is associated with the diagnosis and also prognosis of CRS [4,32,33]. Echocardiography may carry prognostic value in patients with CRS. In a cohort of 30,681 patients, including 2512 patients with CRS, an increasing PA pressure and higher RV diameter were independently associated with a higher incidence of CRS [34]. Regarding kidney imaging, renal ultrasonography, namely intrarenal venous flow, is associated with the prognosis of patients with CRS [35]. Another limitation is that only in-hospital events and codes were included in this analysis. However, it is plausible that all patients with such events are managed in hospitals.

## 5. Conclusions

In conclusion, our unsupervised analysis identified statistically driven groups of patients with different phenotypes but similar prognoses, which supports the classification of CRS based on the vascular aspect of pathophysiology, rather than the chronology of heart and kidney failure. This approach could help to individualize medical treatment.

## Figures and Tables

**Table 1:** Baseline characteristics of patients with CRS according to patient clusters.

	Cluster 1	Cluster 2	Cluster 3	Cluster 4	Total
	(*n* = 1930)	(*n* = 2487)	(*n* = 2163)	(*n* = 7085)	(*n* = 13,665)
Age (years), mean ± SD	76.4 ± 9.9	81.7 ± 8.6	73.9 ± 9.9	82.4 ± 9.7	80.1 ± 10.2
Age ≥ 75 yrs, *n* (%)	1168 (60.5)	2111 (84.9)	1071 (49.5)	6109 (86.2)	10,459 (76.5)
Sex (male), *n* (%)	1490 (77.2)	1643 (66.1)	1199 (55.4)	3472 (49.0)	7804 (57.1)
Hypertension, *n* (%)	1752 (90.8)	2059 (82.8)	2056 (95.1)	5343 (75.4)	11,210 (82.0)
Diabetes mellitus, *n* (%)	1166 (60.4)	825 (33.2)	1876 (86.7)	2199 (31.0)	6066 (44.4)
Smoker, *n* (%)	247 (12.8)	280 (11.3)	271 (12.5)	275 (3.9)	1073 (7.9)
Dyslipidemia, *n* (%)	1094 (56.7)	949 (38.2)	1453 (67.2)	1226 (17.3)	4722 (34.6)
Obesity, *n* (%)	455 (23.6)	348 (14.0)	1331 (61.5)	1113 (15.7)	3247 (23.8)
Alcohol consumption, *n* (%)	108 (5.6)	121 (4.9)	170 (7.9)	380 (5.4)	779 (5.7)
Atrial fibrillation, *n* (%)	737 (38.2)	1633 (65.7)	909 (42.0)	3969 (56.0)	7248 (53.0)
Previous pacemaker or ICD, *n* (%)	309 (16.0)	1003 (40.3)	283 (13.1)	1332 (18.8)	2927 (21.4)
Cardiac valve disease, *n* (%)	269 (13.9)	938 (37.7)	540 (25.0)	1605 (22.7)	3352 (24.5)
Dilated cardiomyopathy, *n* (%)	241 (12.5)	663 (26.7)	707 (32.7)	1474 (20.8)	3085 (22.6)
Coronary artery disease, *n* (%)	1809 (93.7)	2131 (85.7)	1462 (67.6)	2254 (31.8)	7656 (56.0)
Previous MI, *n* (%)	683 (35.4)	368 (14.8)	99 (4.6)	30 (0.4)	1180 (8.6)
Previous PCI, *n* (%)	744 (38.5)	162 (6.5)	177 (8.2)	91 (1.3)	1174 (8.6)
Previous CABG, *n* (%)	62 (3.2)	57 (2.3)	77 (3.6)	36 (0.5)	232 (1.7)
Peripheral artery disease, *n* (%)	1534 (79.5)	1948 (78.3)	1002 (46.3)	787 (11.1)	5271 (38.6)
Ischemic stroke, *n* (%)	112 (5.8)	122 (4.9)	79 (3.7)	217 (3.1)	530 (3.9)
Intracranial bleeding, *n* (%)	26 (1.3)	35 (1.4)	22 (1.0)	118 (1.7)	201 (1.5)
Lung disease, *n* (%)	464 (24.0)	999 (40.2)	643 (29.7)	1999 (28.2)	4105 (30.0)
Liver disease, *n* (%)	80 (4.1)	168 (6.8)	137 (6.3)	481 (6.8)	866 (6.3)
Thyroid diseases, *n* (%)	163 (8.4)	551 (22.2)	357 (16.5)	1118 (15.8)	2189 (16.0)
Anemia, *n* (%)	448 (23.2)	1177 (47.3)	785 (36.3)	2318 (32.7)	4728 (34.6)
Previous cancer, *n* (%)	582 (30.2)	468 (18.8)	185 (8.6)	1093 (15.4)	2328 (17.0)
Poor nutrition, *n* (%)	129 (6.7)	526 (21.1)	164 (7.6)	1073 (15.1)	1892 (13.8)
Cognitive impairment, *n* (%)	119 (6.2)	322 (12.9)	130 (6.0)	1143 (16.1)	1714 (12.5)
Frailty index, mean ± SD	13.8 ± 9.5	15.4 ± 9.9	15.0 ± 10.1	15.0 ± 9.8	14.9 ± 9.9
Frailty index > 13, *n* (%)	879 (45.5)	1302 (52.4)	1089 (50.3)	3570 (50.4)	6840 (50.1)

Values are *n* (%) or mean ± SD. CABG = coronary artery bypass graft; CKD = chronic kidney disease; COPD = chronic obstructive pulmonary disease; ICD = implantable cardioverter defibrillator; MI = myocardial infarction; PCI = percutaneous coronary intervention; SD = standard deviation.

**Table 2:** Hazard ratios (95% CI) associated with cardiorenal syndrome clusters (vs. cluster 1) for incident outcomes.

	Unadjusted HR (95% CI)	*p*	Adjusted HR (95% CI)	*p*
All-cause death				
Cluster 2 (vs. Cluster 1)	1.73 (1.62–1.85)	<0.0001	1.40 (1.31–1.50)	<0.0001
Cluster 3 (vs. Cluster 1)	0.89 (0.82–0.95)	0.001	1.01 (0.94–1.09)	0.75
Cluster 4 (vs Cluster 1)	1.49 (1.40–1.58)	<0.0001	1.20 (1.13–1.28)	<0.0001
Cardiovascular death				
Cluster 2 (vs. Cluster 1)	1.90 (1.69–2.12)	<0.0001	1.48 (1.32–1.66)	<0.0001
Cluster 3 (vs. Cluster 1)	0.92 (0.82–1.05)	0.21	1.04 (0.92–1.18)	0.53
Cluster 4 (vs. Cluster 1)	1.37 (1.24–1.52)	<0.0001	1.05 (0.94–1.16)	0.39
Rehospitalization for HF				
Cluster 2 (vs. Cluster 1)	1.58 (1.46–1.70)	<0.0001	1.43 (1.33–1.55)	<0.0001
Cluster 3 (vs. Cluster 1)	1.14 (1.06–1.23)	0.001	1.18 (1.10–1.27)	<0.0001
Cluster 4 (vs. Cluster 1)	1.28 (1.20–1.36)	<0.0001	1.17 (1.09–1.25)	<0.0001
Myocardial infarction				
Cluster 2 (vs. Cluster 1)	0.92 (0.71–1.20)	0.55	0.89 (0.69–1.16)	0.40
Cluster 3 (vs. Cluster 1)	1.15 (0.92–1.44)	0.22	1.24 (0.99–1.56)	0.06
Cluster 4 (vs. Cluster 1)	0.86 (0.70–1.05)	0.14	0.86 (0.70–1.06)	0.17
Ischemic stroke				
Cluster 2 (vs. Cluster 1)	1.31 (1.01–1.70)	0.04	1.13 (0.87–1.47)	0.38
Cluster 3 (vs. Cluster 1)	1.00 (0.78–1.29)	0.98	1.01 (0.79–1.31)	0.91
Cluster 4 (vs. Cluster 1)	1.16 (0.94–1.44)	0.17	0.97 (0.78–1.22)	0.82
Dialysis or renal transplantation				
Cluster 2 (vs. Cluster 1)	0.79 (0.68–0.92)	0.003	0.99 (0.85–1.16)	0.93
Cluster 3 (vs. Cluster 1)	1.31 (1.16–1.49)	<0.0001	1.33 (1.17–1.51)	<0.0001
Cluster 4 (vs. Cluster 1)	0.80 (0.71–0.90)	<0.0001	1.04 (0.92–1.17)	0.52

HR = hazard ratio; Adjusted HR = age- and sex-adjusted.

## Data Availability

The data can be shared on reasonable request.

## Appendix A

**Table A1:** ICD10 codes.

Comorbidity or Medical History	Codes
AF management care	I48
Strokes	
Ischaemic stroke	I63, I66, I67
Stroke, unspecified	I64
Haemorrhagic stroke	I60–I62, I69
Ischaemic heart disease	I20–I25
Heart failure	I50, I110, I130, I132, I131, I139
Valvular disease	I05–I091, I33–I39, Q22, Q23
Hypertension	I10–I15
Diabetes mellitus	E10–E14
Vascular diseases	
Myocardial infarction	I21, I252
Peripheral arterial disease	I70–I73
Occlusions	I65, I77
Obesity	E65–E66
Chronic kidney disease	N17–N19 (+N28) codes for renal insufficiency, dialysis (Z49, Z992), E102, I12, I13 (transplantation (Z940, T861) was excluded)
Liver disease	K70–K77, procedures for liver transplantation or resection
Dyslipidaemia	E78
Anaemia	D50–D64
Lung disease	J40–J70, J961
Including emphysema and chronic obstructive pulmonary disease	J43, J44
Alcohol-related diagnoses	E244, F10, G312, G621, G721, I426, K292, K70, K860, O354, P043, Q860, T51, Y90, Y91, Z502, Z714
Cancer within preceding 5 years	Entire C-series

**Table A2:** Incident outcomes according to the type of cardiorenal syndrome cluster.

	Cluster 1	Cluster 2	Cluster 3	Cluster 4
All-cause death	1362 (20.2)	2158 (39.5)	1468 (17.6)	5811 (32.5)
Cardiovascular death	476 (7.1)	837 (15.3)	534 (6.4)	1888 (10.6)
Rehospitalization for heart failure	1222 (29.7)	1624 (55.4)	1569 (34.5)	4328 (42.2)
Myocardial infarction	132 (2.0)	100 (1.9)	187 (2.3)	302 (1.7)
Ischemic stroke	110 (1.7)	118 (2.2)	137 (1.7)	343 (2.0)
Dialysis or renal transplantation	397 (6.7)	282 (5.5)	616 (8.8)	886 (5.5)
